# Confirmation value of Western blotting in detecting anti-treponema pallidum specific antibodies with suspicious results

**DOI:** 10.1186/s40001-022-00633-y

**Published:** 2022-02-02

**Authors:** Siqi Xu, Hongsheng Li, Xiaoyan Wu, Jianwei Guo, Jiaoli Zhang, Xuqi Hu

**Affiliations:** The Second Hospital of Jiaxing, No. 1518, Huancheng North Road, Nanhu District, Jiaxing, 314000 Zhejiang China

**Keywords:** ELISA, TPPA, Treponema pallidum antibody, Western blotting

## Abstract

**Background:**

Due to the inconsistent results of anti-treponema pallidum (TP) specific antibodies by enzyme-linked immunosorbent assay (ELISA) and *Treponema pallidum* granule agglutination assay (TPPA) in clinical work, there will be a certain proportion of false-positives and false-negatives depending on TPPA as confirmation results. This study aimed to evaluate the necessity of Western blotting (WB) in samples with inconsistent results in detecting anti-TP antibodies by ELISA and TPPA.

**Methods:**

Specific anti-TP test results in our clinical laboratory were retrospectively analyzed. The specimens with a positive or a negative result, but with colored ELISA plates, were retested by TPPA. WB was used to confirm the suspicious results between ELISA and TPPA. The Chi-square test was used to analyze whether the difference was statistically significant.

**Results:**

A total of 106,757 anti-TP specimens were screened by ELISA from August 2018 to December 2019; 3972 were retested by TPPA, and 3809 were positive by TPPA. ELISA and TPPA showed different results in 163 specimens. Among them, 29 specimens were negative and 134 were positive by ELISA; 76 were negative, 23 were positive, and 64 were “reserve” by TPPA; 93 were negative, 31 were positive, and 39 were suspicious by the WB confirmation test. Compared with WB, the difference in the results of ELISA and TPPA was statistically significant.

**Conclusions:**

TPPA is an effective retest method for anti-TP antibody detection. If the results of anti-TP antibodies by ELISA and TPPA are inconsistent, it is necessary to use WB for confirmation.

*Trial registration* This retrospective analysis is in accordance with the ethical guidelines of China and approved by the second hospital of Jiaxing (jxey-2018048).

## Background

Syphilis is a sexually transmitted disease caused by *Treponema pallidum* (TP). According to official data (http://www.nhfpc.gov.cn/), the cases of syphilis in China continue to increase every year, with syphilis being at the top of the list of sexually transmitted diseases. The diagnosis of syphilis depends on the laboratory serological tests, such as specific enzyme-linked immunosorbent assay (ELISA), chemiluminescence assay (CIA), *Treponema pallidum* particle agglutination assay (TPPA), Western blotting (WB), and nonspecific toluidine red unheated serum assay (TRUST) [1]. Compared with TPPA, ELISA/CIA has higher sensitivity in detecting anti-TP antibodies, and TPPA has higher specificity. Combining the two detection methods can greatly improve the diagnostic sensitivity and specificity; TPPA is often used as a classic method for diagnosing syphilis [2–4]. However, false-negatives can occur with TPPA because of the defects in coating and antigen selection, and the subjective judgment of results [5]. It has also been reported that a certain percentage of biological false positives can be detected with TPPA [5–7]. In this study, samples with inconsistent results of preliminary screening of anti-TP antibodies by ELISA and retest by TPPA were taken as experiment subjects for other WB tests. WB was used to confirm the results of anti-TP antibodies.

## Methods

### Materials

#### Regents

WB was performed for detecting anti-TP antibodies using a TP IgG antibody detection kit and a TP IgM antibody detection kit (Oumeng Diagnostics Ltd., Germany). TPPA was performed using a TP antibody detection kit (Fuji Biological Products Co., Ltd., Japan). ELISA was performed using a TP antibody detection kit (Beijing Wantai Biological Pharmaceutical Co. Ltd., China). TRUST was performed using an anti-TP antibody detection kit (Beijing Wantai, Chian). All reagents are used following the manufacturer’s instructions.

#### Instruments

The following instruments were used in the study: Tecan fully automatic enzyme analyzer (Tecan, Switzerland); Freedom EVOlyzer (Tecan, Switzerland); Oumeng automatic WB analyzer (Oumeng, Germany); EURO Blotmaster II (Oumeng, Germany); Oumeng scanner (Oumeng, Germany) and EUROLine Camera (Oumeng, Germany). All instruments are used following the manufacturer’s instructions.

### Methods

#### Samples

The outpatients and inpatients in our hospital underwent the examination of anti-TP antibodies. The serum samples with inconsistent ELISA and TPPA results from August 2018 to December 2019 were collected, including the negative ELISA and positive or reserved TPPA (ELISA^−^/TPPA^+^ and ELISA^−^/TPPA^reserved^) and the positive ELISA and negative or reserved TPPA (ELISA^+^/TPPA^−^ and ELISA^+^/TPPA^reserved^). The samples were cryopreserved at –70 ℃. A total of 163 samples were collected and thawed for WB-IgM and WB-IgG tests.

#### ELISA test

The ELISA results were interpreted following the instructions of the Wantai TP antibody detection kit. When the sample absorbance value < critical value (usually expressed as sample absorbance value/critical value < 1, that is, S/CO < 1), the anti-TP antibody result is negative; when the sample absorbance value ≥ critical value (S/CO ≥ 1), the anti-TP antibody result is positive. The specimens with a positive, or a negative result, but with colored ELISA plates were retested by TPPA.

#### TPPA test

The TPPA results were interpreted following the instructions of the Fuji TP antibody detection kit. When the reaction image of the unsensitized particles (the final dilution is 1:40) was determined as (−) and the reaction image of the sensitized particles (the final dilution is 1:80) was determined as (+), the TPPA result was considered as positive; regardless of the reaction image of the unsensitized particle, as long as the reaction image of the sensitized particle (the final dilution ratio is 1:80) was determined as (−), the TPPA result was considered as negative; when the reaction image of the unsensitized particles (the final dilution is 1:40) was determined as (–) and the reaction image of the sensitized particles (the final dilution is 1:80) was determined as (±), the TPPA result was considered as "reserved". WB was performed on the samples whose ELISA and TPPA results were inconsistent.

#### WB test

The WB results were interpreted following the instructions of the Oumeng TP antibody detection kit. (1) IgG antibody: no specific antigen band staining was interpreted as negative; one specific antigenic band staining was interpreted as suspicious; more than one specific band staining was interpreted as positive. (2) IgM antibody: no specific antigen band staining was interpreted as negative; one specific antigenic band weak staining was interpreted as suspicious; at least one specific antigenic band staining was interpreted as positive. Single- or double-positive results with WB-IgM and WB-IgG in the same sample were considered as WB “positive”, single or double suspicious results with WB-IgM and WB-IgG were considered as WB "suspicious", and both negative results with WB-IgM and WB-IgG were considered as WB “negative”.

#### Statistical method

SPSS 19.0 statistical software was used to analyze whether the difference in results was found to be statistically significant using the Chi-square test.

## Results

### Basic information of serum samples

From August 2018 to December 2019, 3972 of the 106,757 samples were detected with anti-TP antibodies by ELISA were initially positive (S/CO ≥ 1) or negative (S/CO < 1 but showed chromogeny on the ELISA plate). The TPPA retest showed that 3809 samples were positive. The results of ELISA and TPPA were inconsistent in 163 serum samples, accounting for 4.1% (163/3 972) of the total retest samples. Of 163 samples, 29 were ELISA^−^/TPPA^+^ or ELISA^−^/TPPA^reserved^, accounting for 17.8% (29/163); and 134 were ELISA^+^/TPPA^−^ or ELISA^+^/TPPA^reserved^, accounting for 82.2% (134/163). The patients included 101 men (61.96%) and 62 women (38.04%). The age of patients ranged from 4 days to 89 years (53.6 ± 17.5). As shown in Table 1, anti-TP antibodies were detected by ELISA and TPPA, and the difference in results was statistically significant (*P* < 0.05).

**Table 1:** Samples’ results detected by ELISA and TPPA method

Method	Result	ELISA		Total number	The value of χ^2^/P
		Positive	Negative		
TPPA	Positive	3809	22	3834	103,831.414/0.000
	Negative	76	102,785	102,861	
	Reserve	58	7	65	
Total number		3943	102,814	106,757	

### Retest and confirmation results of negative samples by ELISA

As shown in Table 2, among 163 samples with suspicious results of anti-TP antibodies, 29 samples were negative by ELISA (17.8%). Among them, the TPPA retest was positive in 22 cases and reserved in 7 cases; while WB was positive in 13 cases, suspicious in 9 cases, and negative in 7 cases. The S/CO values of the 29 samples ranged from 0.201 to 0.984. The S/CO was < 0.5 in seven samples. The TPPA retest was positive in six cases and reserved in one case, while WB was positive in three cases, suspicious in three cases, and negative in one case. Further, in 22 samples, 0.5 ≤ S/CO < 1. The TPPA retest was positive in 16 cases and reserved in 6 cases, while WB was positive in 10 cases, suspicious in 6 cases, and negative in 6 cases.

**Table 2:** Retest and confirmation results of negative samples by ELISA

Method	Result	The S/CO values by ELISA		Total number
		S/CO < 0.5	0.5 ≤ S/CO < 1	
TPPA	Positive	6	16	22
	Reserve	1	6	7
WB	Negative	1	6	7
	Positive	3	10	13
	Suspicious	3	6	9

### Retest and confirmation results of positive samples by ELISA

As shown in Table 3, among 163 samples with suspicious results of anti-TP antibodies, 134 samples were positive by ELISA (17.8%). Among them, the TPPA retest was negative in 76 cases and reserved in 58 cases, while WB was positive in 18 cases, suspicious in 30 cases, and negative in 86 cases. The S/CO values of the 134 samples ranged from 1.01 to 19.15. In 54 samples, 1 ≤ S/CO < 2. The TPPA retest was negative in 42 cases and reserved in 12 cases, while WB was positive in 7 cases, suspicious in 7 cases, and negative in 40 cases. In 32 samples, 2 ≤ S/CO < 3. The TPPA retest was negative in 17 cases and reserved in 15 cases, WB was positive in 1 case, suspicious in 11 cases, and negative in 20 cases. In 27 samples, 3 ≤ S/CO < 5. The TPPA retest was negative in 12 cases and reserved in 15 cases, while WB was positive in 4 cases, suspicious in 8 cases, and negative in 15 cases. In 21 samples, S/CO ≥ 5. The TPPA retest was negative in 5 cases and reserved in 16 cases, while WB was positive in 6 cases, suspicious in 4 cases, and negative in 11 cases.

**Table 3:** Retest and confirmation results of positive samples by ELISA

Method	Result	The S/CO values by ELISA				Total number
		1 ≤ S/CO < 2	2 ≤ S/CO < 3	3 ≤ S/CO < 5	S/CO ≥ 5	
TPPA	Negative	42	17	12	5	76
	Reserve	12	15	15	16	58
WB	Negative	40	20	15	11	86
	Positive	7	1	4	6	18
	Suspicious	7	11	8	4	30

### Comparison of the test results of ELISA and WB in 163 samples

The results of WB-IgM and WB-IgG in 163 samples with different results of ELISA and TPPA were analyzed. As shown in Table 4, among the 29 ELISA-negative samples, WB results were negative in 7 cases, positive in 13 cases, and suspicious in 9 cases. Among 134 ELISA-positive samples, 86 cases were negative, 18 cases were positive, and 30 cases were suspicious, with a specificity of 7.53% (7/93) and a sensitivity of 58.06% (18/31). Significant differences were found between ELISA and WB in detecting anti-TP antibodies (*P* < 0.05).

**Table 4:** Comparison the results of ELISA and WB method in 163 samples

Method	Result	WB			Total number	The value of χ^2^/P
		Negative	Positive	Suspicious		
ELISA	Negative	7	13	9	29	19.800/0.000
	Positive	86	18	30	134	
Total number		93	31	39	163	

### Comparison of the test results of TPPA and WB in 163 samples

The results of WB-IgM and WB-IgG in 163 samples with different results of ELISA and TPPA were analyzed. As shown in Table 5, among the 76 TPPA-negative samples, WB results were negative in 59 cases, positive in 9 cases, and suspicious in 8 cases. Among the 22 TPPA-positive samples, WB results were negative in 2 cases, positive in 13 cases, and suspicious in 7 cases, with a specificity of 63.44% (59/93) and a sensitivity of 41.94% (13/31). Significant differences were found between TPPA and WB in detecting anti-TP antibodies (*P* < 0.05).

**Table 5:** Comparison of the results of TPPA and WB method in 163 samples

Method	Result	WB			Total number	The value of χ^2^/P
		Negative	Positive	Suspicious		
TPPA	Negative	59	9	8	76	47.621/0.000
	Positive	2	13	7	22	
	Reserved	32	9	24	65	
Total number		93	31	39	163	

## Discussion

According to the diagnosis and treatment guidelines of syphilis infection in our country [1], we cannot diagnose syphilis without laboratory examination, especially the serological test of TP, whether it is congenital syphilis or late syphilis, or early syphilis or late syphilis. It was found that a certain proportion of samples were negative for ELISA and positive or reserved for TPPA, or positive for ELISA and negative or reserved for TPPA when using ELISA for primary screening and TPPA for the retest of anti-TP antibodies. Since TPPA is not a true diagnostic method, Chinese Center for Disease Control and Prevention (CDC) points out that when anti-TP antibody test results are inconsistent with TPPA test results, WB is recommended for the confirmation of anti-TP antibody results (http://www.ncstdc.org/). WB is also recommended by the European CDC as a confirmation test for biological false positives; it is regarded as the gold standard for anti-TP antibody detection [8]. Compared with other anti-TP-specific antibody assays, WB has the best sensitivity and specificity [9–11]. In this study, WB was introduced to confirm the samples with inconsistent results between ELISA and TPPA.

Wangxinyu [12] pointed that when the results of TP-antibody test are weak reactivity, TPPA should not be used as the basis of clinical diagnosis, so as to avoid missed diagnosis and misdiagnosis; WB is used as the diagnosis experiment of syphilis, of which suspicious positive results are common. Those results were consistent with our results. Regardless of the experimental population, method and region, researchers all agreed to use WB as the confirmation method of TP-specific antibody detection [11–13].

In this study, the results of ELISA and TPPA were inconsistent with 163 serum samples. The age of patients was 53.6 ± 17.5 years, including 62 elderly patients (≥ 60 years old), accounting for 38.03%. Among the 29 negative ELISA samples, 22 were confirmed positive by WB, and the lowest S/CO was 0.232. Therefore, in clinical work, special attention should be paid to the samples with S/CO < 1, and the chromogenic enzyme plate should be visible to the naked eye. Confirmed by WB, 93 cases (57.06%) were negative, 31 cases (19.02%) were positive, and 39 cases (23.93%) were suspicious. The 39 anti-TP antibody samples with a suspicious result by WB were retrospectively followed up. Among them, two samples (hemodialysis patients) turned negative, five samples turned positive, and no follow-up test data were found for the remaining samples. This indicated that most of the samples with inconsistent ELISA and TPPA results were negative for diagnosis by WB. When the WB results were still suspicious, the traceable data showed that most samples would turn positive.

Samples with inconsistent TPPA and WB results and all reserved results by TPPA were queried in the laboratory information system. A total of 84 samples were queried for the results of anti-nuclear antibodies, tumor markers, rheumatoid factors, and the use of immunoglobulin. Immunoglobulins were not used in all 84 samples. Further, 62 samples of tumor markers and other laboratory test results were within the reference range or could not be queried. Among the remaining 22 samples, 13 samples had different degrees of increased ferritin level, accounting for 59.09% (13/22). Five samples showed elevated levels of prostate cancer-specific antigen (PSA). Five samples had different degrees of Squamous Cell Carcinoma Antigen antigen (SCC) elevation, and some samples also had ferritin or PSA elevation. In addition, the levels of carbohydrate antigen 12-5 (CA12-5), CA15-3, CA724, CA242, carcinoembryonic antigen (CEA), and anti-nuclear antibodies increased in individual samples. The available data showed that most of the samples had different degrees of increase in the levels of tumor markers, indicating that they might interfere with the detection of anti-TP antibodies to a certain extent.

Compared with the results of ELISA and TPPA, the difference was statistically significant, indicating the necessity of using TPPA to review the test results of primary screening samples in clinical work. Using samples with inconsistent ELISA and TPPA results as objects, the differences between ELISA and WB results and those between TPPA and WB results were found to be statistically significant, indicating that the use of WB for anti-TP antibody detection confirmation test was very important.

WB has a certain percentage of “suspicious” results; however, the tracking data show that most of them would turn positive. Most of the suspected positive results of anti-TP antibodies could be confirmed by WB, so as to provide a more convincing test report for the diagnosis by clinicians.

## Conclusions

For the samples with inconsistent results of TP-antibody by ELISA and TPPA, TPPA should not be used as a confirmation test for diagnosis, further detection by WB method is very necessary and important.

## Data Availability

The datasets generated and/or analyzed during the current study are available from the corresponding author on reasonable request.

## Abbreviations

WB: Western blotting
TP: *Treponema pallidum*
ELISA: Enzyme-linked immunosorbent assay
TPPA: *Treponema pallidum* Granule agglutination assay
CIA: Chemiluminescence assay
S/CO: Sample absorbance value/critical value
CDC: Center for Disease Control and Prevention
PSA: Prostate specific antigen
SCC: Squamous Cell Carcinoma Antigen antigen
CA12-5: Carbohydrate antigen 12-5
CA15-3: Carbohydrate antigen 15-3
CA724: Carbohydrate antigen 724
CA242: Carbohydrate antigen 242
CEA: Carcinoembryonic antigen
